# Efficacy of chlorine solutions used for hand hygiene and gloves disinfection in Ebola settings: a systematic review

**DOI:** 10.1186/2047-2994-4-S1-O13

**Published:** 2015-06-16

**Authors:** J Hopman, Z Kubilay, T Allen, H Edrees, D Pittet, B Allegranzi

**Affiliations:** 1 Medical Microbiology/Unit Infection control, Radboud University Medical Center, Nijmegen, Netherlands; 2 Service Delivery and Safety, Health Systems and Innovation (HIS), WHO, Geneva, Switzerland; 3 Library, WHO, Geneva, Switzerland; 4 Faculty of Medicine, University of Geneva Hospitals, Geneva, Switzerland

## Introduction

Chlorine solutions (CS) have been widely used for hand hygiene (HH) in the West African countries affected by the current Ebola outbreak. However, no HH guidelines recommend the use of CS for HH practices.

## Objectives

To conduct a systematic review to assess the comparative efficacy of CS for HH or disinfecting gloves vs alcohol-based handrubs or other antisepsis products.

## Methods

PubMed (including MEDLINE) and Ovid EMBASE databases were searched on 26th September 2014 with no time, age, human, language or geographical restrictions. The reference lists of relevant articles were also screened.

## Results

Out of 1931 hits, no study was found on the efficacy of CS for HH or glove disinfection to reduce the transmission of filovirus or any enveloped viruses among health workers. Four articles about the efficacy of sodium hypochlorite to reduce bacterial count on hands of healthy volunteers in a laboratory setting were selected. In these studies, different concentrations of CS were used: an aqueous solution of sodium hypochlorite (Milton 1:80); a 4% sodium hypochlorite solution; 0.5% bleach; and microfiber releasing 400 ppm bleach. Many different comparators were tested, the most common being water and soap, 2% or 4% chlorhexidine gluconate, alcohol (60% isopropanol or ethanol). Different contact times (from 10 seconds up to 5 minutes) were applied for both the CS and the comparators. These studies yielded conflicting results, some showing higher efficacy of CS and others inferiority. No study was found on efficacy of CS for glove disinfection.

## Conclusion

Overall, there is very limited and very low-quality evidence to evaluate the efficacy of CS in comparison to other agents for HH and no evidence exists for glove disinfection. No manuscript evaluating the efficacy of sodium hypochlorite for (enveloped) viruses was found. In the included studies, differences in CS concentrations, contact time and microorganisms were observed. The WHO guidelines developed based on this review suggest that CS may be used in emergency situations, but strongly recommend implementing strategies to change to alcohol-based handrubs or soap and water.

## Disclosure of interest

None declared.

